# Pathogenesis and transmissibility of highly (H7N1) and low (H7N9) pathogenic avian influenza virus infection in red-legged partridge (*Alectoris rufa*)

**DOI:** 10.1186/1297-9716-42-24

**Published:** 2011-02-07

**Authors:** Kateri Bertran, Elisa Pérez-Ramírez, Núria Busquets, Roser Dolz, Antonio Ramis, Ayub Darji, Francesc Xavier Abad, Rosa Valle, Aida Chaves, Júlia Vergara-Alert, Marta Barral, Ursula Höfle, Natàlia Majó

**Affiliations:** 1 Centre de Recerca en Sanitat Animal (CReSA), UAB-IRTA, Campus de la Universitat Autònoma de Barcelona, 08193 Bellaterra, Barcelona, Spain; 2 Instituto de Investigación en Recursos Cinegéticos, IREC (CSIC, UCLM, JCCM), Ronda de Toledo s/n, 13071 Ciudad Real, Spain; 3 Departament de Sanitat i Anatomia Animals, Universitat Autònoma de Barcelona, 08193 Bellaterra, Barcelona, Spain; 4 NEIKER-Instituto Vasco de Investigación y Desarrollo Agrario, Departmento de Sanidad Animal, Berreaga 1, 48160 Derio, Bizkaia, Spain

## Abstract

An experimental infection with highly pathogenic avian influenza virus (HPAIV) and low pathogenic avian influenza virus (LPAIV) was carried out in red-legged partridges (*Alectoris rufa*) in order to study clinical signs, gross and microscopic lesions, and viral distribution in tissues and viral shedding. Birds were infected with a HPAIV subtype H7N1 (A/Chicken/Italy/5093/1999) and a LPAIV subtype H7N9 (A/*Anas crecca*/Spain/1460/2008). Uninoculated birds were included as contacts in both groups. In HPAIV infected birds, the first clinical signs were observed at 3 dpi, and mortality started at 4 dpi, reaching 100% at 8 dpi. The presence of viral antigen in tissues and viral shedding were confirmed by immunohistochemistry and quantitative real time RT-PCR (qRRT-PCR), respectively, in all birds infected with HPAIV. However, neither clinical signs nor histopathological findings were observed in LPAIV infected partridges. In addition, only short-term viral shedding together with seroconversion was detected in some LPAIV inoculated animals. The present study demonstrates that the red-legged partridge is highly susceptible to the H7N1 HPAIV strain, causing severe disease, mortality and abundant viral shedding and thus contributing to the spread of a potential local outbreak of this virus. In contrast, our results concerning H7N9 LPAIV suggest that the red-legged partridge is not a reservoir species for this virus.

## Introduction

In recent years, avian influenza has become one of the most important challenges that have emerged from animal reservoirs [1,2]. The current outbreaks detected in poultry and wild birds in many Asian, European and African countries are of concern not only to the poultry industry, in which they produce an economically devastating disease, but also to public health [3]. The potential of these viruses to cause a pandemic represents a constant threat to poultry, wild birds and humans worldwide, underlining the importance of avian reservoirs for any subtype of avian influenza virus. The epidemiology of avian influenza viruses is complex, and there are still many unknown aspects, especially in relation to the reservoir. Wild birds, particularly those belonging to the orders *Anseriformes *and *Charadriiformes*, have long been recognised as the natural reservoir for influenza A viruses [4]. Since its first isolation from wild birds in 1961, influenza A viruses have been isolated from 105 wild bird species belonging to 26 families [3,5].

Some studies suggest that turkeys, pheasants, and Japanese quails are more susceptible than chickens to infection by avian influenza viruses transmitted from free-living aquatic birds [6,7]. Experimental infections have shown that highly pathogenic avian influenza virus (HPAIV) can cause specific clinical signs and mortality in the above mentioned species [8], and that pheasants are efficient shedders of low pathogenic avian influenza virus (LPAIV) [6]. Furthermore, open range raising of birds has been identified as one of the factors contributing to the increase of avian influenza virus outbreaks and their impact [3]. Nevertheless, to date, most experimental studies on avian influenza are based on either chickens, turkeys or waterfowl species, while investigation into the ability of influenza A viruses to replicate in minor poultry species is scarce [6,8,9], and numerous aspects of the epidemiology of both LPAIV and HPAIV in free-range raised poultry and game birds still remain unclear.

Surprisingly, there are no studies about the susceptibility to infection and the pathogenicity of avian influenza in red-legged partridge (*Alectoris rufa*). This important game bird species is widely distributed in south-western Europe and in the south of England. During the last decades, natural populations of this game bird have declined in most of its distribution range [10]. The strategy of many hunting estate managers to overcome the lack of wild partridges has been the release of farm-reared birds. Red-legged partridges are raised in outdoor operations that are abundant in Spain, comprising currently 7% of the global avian production system [11]. Although some authors have stated that every year, between 3 and 4.5 million of farm reared red legged partridges are released into the wild [12], considering recent information from hunters, farms, hunting estates and numbers of captures, the real number of partridges released in Spain could be quite close to 10 million per hunting season [13]. The lack of adequate biosecurity measures in part of the red-legged partridge farms, together with limited sanitary control measures prior to and after release into the wild, could favour the introduction, adaptation, maintenance, and spread of pathogens including avian influenza (AI) viruses.

In the present study, an experimental infection with both LPAIV and HPAIV was carried out in red-legged partridges in order to determine clinical signs, gross and microscopic lesions. Viral distribution in tissues and the extent and duration of viral shedding were also evaluated by means of qRRT-PCR and immunohistochemistry. In addition, the ability of effective transmission among animals was also assessed.

## Materials and methods

### Viruses

For the present study, two strains of avian influenza virus were used. An HPAIV H7N1 subtype isolate (A/Chicken/Italy/5093/1999) was kindly provided by Dr Ana Moreno from the Istituto Zooprofilattico Sperimentale della Lombardia e dell'Emilia Romagna (IZSLER). A LPAIV H7N9 subtype isolate (A/*Anas crecca*/Spain/1460/2008) was obtained from the ongoing surveillance program carried out in Catalonia, north-east Spain. Designations hereafter will be H7N1 for the A/Chicken/Italy/5093/1999 virus and H7N9 for the A/*Anas crecca/*Spain/1460/2008 virus. The amino acid sequences at the HA0 cleavage site were PEIPKGSRVRR*GLF for the isolate H7N1 and PEIPKGR/GLF for the isolate H7N9.

Stocks of avian influenza viruses were produced in 9-day-old embryonated specific pathogen free (SPF) chicken eggs, by a sixth passage in the H7N1 strain and by a first passage in the H7N9 strain. In both cases, the allantoic fluid was harvested at 48 hours post inoculation, aliquoted and stored at -80°C until use. Virus was diluted tenfold in phosphate buffer saline (PBS) for titration in 9-day-old embryonated chicken eggs. The 50% egg lethal dose (ELD) for H7N1 subtype, and the 50% egg infective dose (EID) for H7N9 subtype, were determined using the Reed and Muench method [14]. The H7N1 subtype demonstrated an intravenous pathogenicity index (IVPI) of 3.0 [15] and showed an amino acid sequence in the cleavage site characteristic of HPAIV [16].

### Animals

Fifty-six red-legged partridges of two months of age were used in this study. Male and female birds were included in approximately equal numbers. The animals were raised in the experimental farm of Instituto de Investigación en Recursos Cinegéticos (IREC), where serum samples were collected and tested, prior to inoculation, to ensure that birds were serologically negative for avian influenza virus by a competition ELISA test (ID-VET, Montpellier, France) and a specific hemagglutination inhibition (HI) test for the H7 subtype. Upon arrival at the Centre de Recerca en Sanitat Animal (CReSA), the animals were housed in biosafety level 3 (BSL-3) facilities. The partridges were kept one week for acclimation, and then they were randomly assigned to experimental groups and housed separately in negative-pressured isolators with HEPA-filtered air. Food and water were provided *ad libitum *throughout the experiment.

### Experimental design

Fifty-six birds were separated into five groups. For each virus, the partridges were subdivided into two experimental groups composed of twelve partridges. Groups A (1A and 2A) were used to evaluate the mortality and transmissibility of the viruses, as well as the virus shedding pattern. Groups B (1B and 2B) were used for pathological studies. Both groups infected with the HPAIV subtype (groups 1A and 1B) were inoculated intranasally with 10^6 ^ELD_50 _of the H7N1 strain. In group 1A, 4 out of 12 partridges were not infected but placed into the isolator with the inoculated birds one hour after inoculation; these uninfected birds were referred to as contacts. Both groups infected with the LPAIV subtype (groups 2A and 2B) were inoculated intranasally with 10^5 ^ELD_50 _of the H7N9 strain. As in the case of group 1A, in group 2A four contacts were included that were not infected but placed into the isolator with the inoculated birds one hour after inoculation. A fifth group (3) of eight partridges was used as control; these birds were inoculated intranasally with PBS solution. All procedures were performed according to the requirements of the Ethics Committee of Animal and Human Experimentation of the Universitat Autònoma de Barcelona.

### Sampling

All birds were monitored daily for clinical signs and scored following the OIE system [17]: healthy (0), sick (1), severely sick (2), moribund or dead (3). Since this is a subjective clinical assessment, "sick" birds would be the ones showing one of the following signs, and "severely sick" more than one of the following signs: respiratory involvement, depression, diarrhoea, cyanosis of the exposed skin or wattles, oedema of the face and/or head, nervous signs. Every day during the first 10 days post infection (dpi), and also at 12 dpi and 15 dpi, oropharyngeal and cloacal swabs, and feather pulp samples were obtained from partridges of groups 1A and 2A in order to measure viral shedding. The same samples were collected at 3, 6, 10 and 15 dpi from the control group. Mortality and mean death time (MDT) were calculated from these three groups. At 3, 6, 10 and 15 dpi, three animals of groups 1B and 2B, and two animals of the control group, were euthanised. All euthanised and naturally dead partridges were necropsied to evaluate gross lesions and obtain samples for pathological studies. Blood samples were collected in tubes without anticoagulant at 0, 6, 8, 10 and 15 dpi from those animals ethically euthanised. Samples collected for detection of viral shedding and serum samples were stored at -80°C until use.

### Histopathology

Necropsies and tissue sampling were performed according to a standard protocol. After fixation in 10% neutral buffered formalin and embedding in paraffin, tissue sections were processed routinely for haematoxylin/eosin (H/E) staining. The following tissues were examined: oesophagus, crop, proventriculus, gizzard, duodenum, jejunum-ileum, caecum/cecal tonsil, colon, rectum, pancreas, liver, kidney, adrenal gland, gonad, nasal turbinates, trachea, lung, heart, breast muscle, skin, bone marrow, spleen, bursa of Fabricius, thymus, brain, spinal cord and sciatic nerve.

### Avian influenza virus detection by immunohistochemistry (IHC)

An immunohistochemical technique based on Avidin-biotin complex immunoperoxidase (ABC) system was performed as previously described [18,19]. The primary antibody was a mouse-derived monoclonal commercial antibody against nucleoprotein of influenza A virus (IgG2a, Hb65, ATCC). As a secondary antibody, a biotinylated goat anti-mouse IgG antibody (GaMb, Dako E0433, Glostrup, Denmark), was used. As positive control, tissues previously demonstrated to be positive against nucleoprotein of influenza A virus by IHC were used. Negative controls were tissues from sham-inoculated animals and tissues incubated without the primary antibody. The following score was used in order to measure the staining in tissues: no positive cells (-), single positive cells (+), scattered groups of positive cells (++), widespread positivity (+++).

### Avian influenza virus quantitation by real time RT-PCR (qRRT-PCR)

Viral RNA quantitation using one step qRRT-PCR was carried out in oropharyngeal and cloacal swabs, and feather pulp samples. Viral RNA was extracted with QIAamp viral mini kit (Qiagen, Valencia, CA, USA) and amplified as previously described [20] in Fast7500 equipment (Applied Biosystems, Foster City, CA, USA). A one step qRRT-PCR assay for *M *gene was applied to determine the viral RNA titre, detecting viral RNA genome (vRNA), the copy of vRNA (cRNA) and mRNA. The limit of detection of the technique was 1.46 log_10 _viral RNA copies/sample.

### Serology

A competitive enzyme-linked immunosorbent assay (C-ELISA) test was carried out in order to detect avian influenza antibodies using a commercially available C-ELISA kit (ID-VET, Montpellier, France) performed according to the manufacturer's instructions.

## Results

### Clinical signs

Clinical signs were only observed in H7N1 infected partridges, which showed signs from scores 1 to 3. There were no relevant differences in clinical signs between inoculated and contact animals.

All birds infected with H7N1 showed clinical signs that started at 3 dpi and consisted in depression, apathy and ruffled feathers. Impaired respiration and diarrhoea were observed in some of the animals. At 8 dpi, 3 of the 4 surviving partridges presented severe neurological signs consisting in torticollis, circling, incoordination, leg/wing paralysis, opisthotonus and head tremors while two birds were recumbent and unresponsive. Mortality started at 4 dpi and lasted until 8 dpi. Intranasal inoculation of the H7N1 virus resulted in 100% mortality, and mean death time (MDT) was 6.42 dpi. Birds with neurological signs, together with the two other animals that presented prostration, were euthanised for ethical reasons. No mortality or clinical signs were observed in H7N9 infected partridges, and in the controls.

### Gross findings

**Figure 1:**
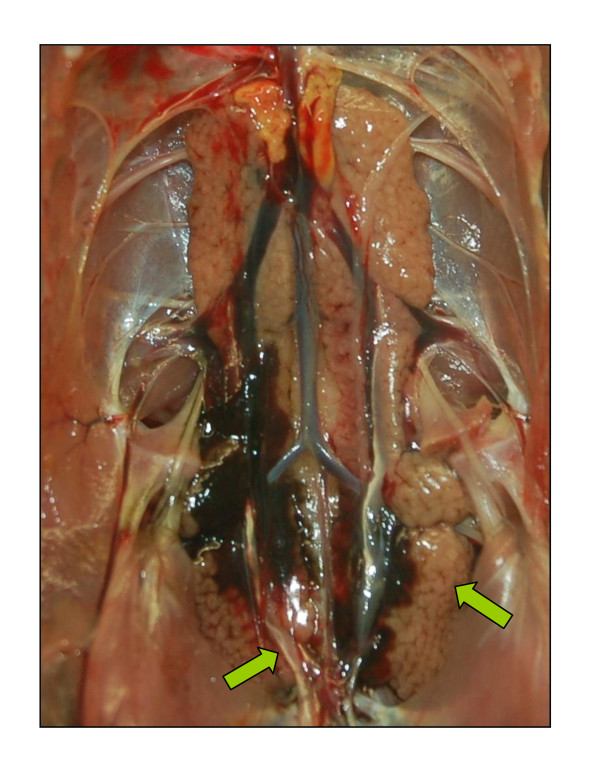
**Kidney lesions of a H7N1 HPAIV (A/Chicken/Italy/5093/1999) infected partridge consisting in parenchymal pallor, lobular surface architecture and urate deposits in the urethers, 6 dpi**.

Lesions associated with influenza were observed only in H7N1 infected partridges from 3 dpi onwards. HPAIV infected partridges, both inoculated and contact, were generally in bad body condition. At 3 dpi, petechial haemorrhages on the *fasciae *sheaths of the muscles of rear legs were seen in some birds, and thymus atrophy was detected until the end of the experiment. Kidney lesions were present from 3 dpi onwards and were characterised by parenchymal pallor and accentuated lobular surface architecture, often accompanied by urate deposits in the urethers (Figure 1). Some partridges showed brain congestion from 6 dpi onwards, and, in most cases, hyperaemic vessels were detected in almost all organs. No lesions were observed in H7N9 infected birds, and birds from the control group.

### Histopathological findings

Histological lesions were only observed in H7N1 infected partridges. The onset of microscopic findings was at 3 dpi, with the lesions being the most intense between 6 dpi and 8 dpi. The most severely affected organs were the kidney, adrenal gland, feather follicles and CNS (brain and spinal cord). Only the gonads, spleen, bone marrow and sciatic nerve did not show significant histopathological changes. No significant lesions were observed in H7N9 inoculated animals, and in the control birds.

**Figure 2:**
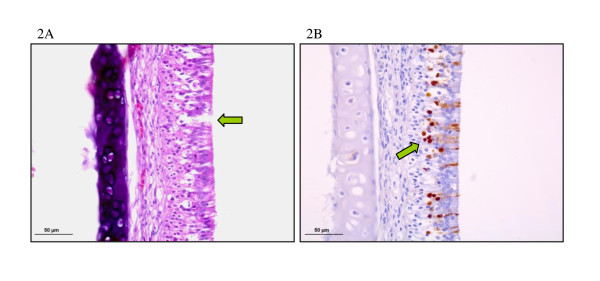
**Nasal turbinates, 6 dpi; (A) Necrosis of single cells of the olfactory epithelium, H/E. (B) Positive staining in olfactory epithelial cells, IHC**.

**Figure 3:**
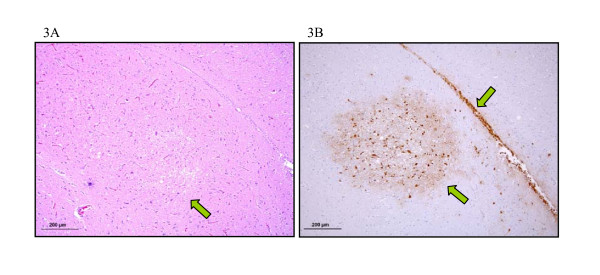
**Brain, 5 dpi;** (**A**) Focal areas of malacia, H/E. (**B**) Positive staining in neurons, ependymal cells and glial cells, IHC.

Lesions in the digestive tract, liver, pancreas, kidney, adrenal gland, myocardium, breast muscle, Bursa of Fabricius and respiratory tract (Figure 2A) were mostly characterised by necrosis and light to moderate heterophilic infiltrates. Necrosis of the epidermal collar epithelial cells, in some cases in association with heterophilic infiltrate, was observed in feather follicles from 6 dpi onwards. In the brain, the most striking finding consisted in multifocal areas of malacia (Figure 3A). Evident necrosis of ependymal cells of the ventricles and epithelial cells of the choroid plexus was present. The cerebellum frequently showed multifocal areas of moderate chromatolysis of Purkinje neurons. Similar lesions were seen in the spinal cord from 6 dpi onwards; multifocal areas of mild spongiosis of the neuropil and neuronal chromatolysis, especially surrounding the medullary canal, were observed. In addition, some animals at 8 dpi showed focal heterophilic inflammatory infiltrate in the leptomeninges.

### Avian influenza virus detection by immunohistochemistry (IHC)

Influenza A viral antigen was only detected in tissues of H7N1 infected partridges. In some organs, virus was more frequently and intensely detected, such as the gizzard, pancreas, kidney, adrenal gland, feather follicles and CNS (brain and spinal cord) (Figures 2B, 3B). Antigenic staining was observed both in parenchymal and endothelial cells; it was nuclear and also often cytoplasmic in distribution. In general, positive staining correlated well with histopathological findings (Table 1).

**Table 1:** Average distribution of nucleoprotein antigen, as determined by immunohistochemistry, in tissues sampled from red-legged partridges (*Alectoris rufa*) intranasally inoculated with A/Chicken/Italy/5093/1999 (H7N1) influenza virus

Tissue	3 dpi	6 dpi	8 dpi	Predominant cell types
Esophagus	-	-	-	-
Crop	-	+	-	Squamous polistratified epithelial cells
Proventriculus	-	+	-	Epithelial cells of the gastric glands
Gizzard	+	++	+	Epithelial cells of the gastric glands, cells of the muscularis externa
Duodenum	-	-	-	-
Jejunum-Ileum	-	-	-	-
Cecum/Cecal tonsil	-	+	-	Epithelial cells of the glands, cells of the muscularis externa
Colon	-	-	-	-
Rectum	-	-	-	-
Pancreas	+	+	+	Acinar cells, endothelial cells
Liver	+	+	+	Kupffer cells, endothelial cells
Kidney	++	+++	++	Tubular epithelial cells, endothelial cells
Adrenal gland	+	+++	+++	Corticotrophic, corticotropic cells
Gonad	-	-	+	Epithelial cells of the oviduct
Nasal turbinates	-	+	+	Olfactory epithelial cells, respiratory epithelial cells, epithelial cells of the infraorbital sinuses, salivary and nasal glands
Trachea	+	-	+	Pseudostratified epithelial cells
Lung	+	+	+	Air capillaries cells, macrophages, endothelial cells
Heart	-	+	+	Myocardyocytes, endothelial cells
Breast muscle	-	+	-	Myocytes, endothelial cells
Skin	-	++	+	Epithelial cells of epidermal collar of feather follicles, endothelial cells of pulp
Bone marrow	-	-	-	-
Spleen	+	+	+	Macrophages, endothelial cells
Bursa of Fabricius	-	+	-	Macrophages, endothelial cells
Thymus	-	-	-	-
Brain	-	++	+++	Neurons, ependymal cells, glial cells
Spinal cord	-	+	++	Neurons, ependymal cells, cells of the leptomeninges
Sciatic nerve	-	-	-	-

- = no positive cells; + = single positive cells; ++ = scattered groups of positive cells; +++ = widespread positivity

### Avian influenza virus quantitation by real time RT-PCR (qRRT-PCR)

**Figure 4:**
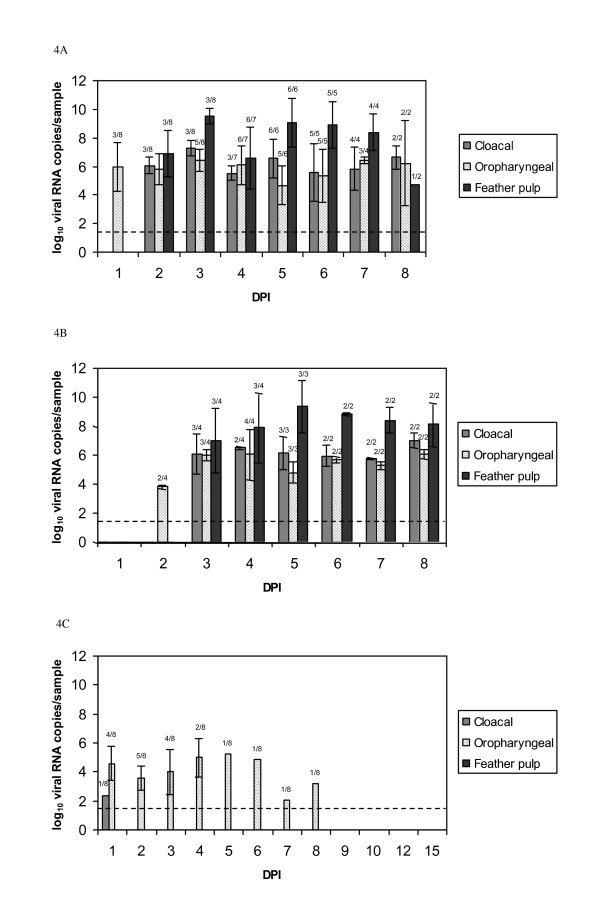
**Viral shedding (expressed as log_10 _viral RNA copies/sample) detected by qRRT-PCR over 8 days in cloacal and oropharyngeal swabs and feather pulp samples of red-legged partridges (*Alectoris rufa*) infected with A/Chicken/Italy/5093/1999 H7N1 HPAIV and A/*Anas crecca/*Spain/1460/2008 H7N9 LPAIV**. In **A **and **B**, rates above the bars indicate the relation between positive birds and the total number of animals examined. Limit of detection is indicated by the dashed line (1.46 log_10 _viral RNA copies/sample). (**A**) H7N1 HPAIV intranasally inoculated partridges, (**B**) H7N1 HPAIV contact partridges, (**C**) H7N9 LPAIV intranasally inoculated partridges.

qRRT-PCR was performed on oropharyngeal and cloacal swabs, and feather pulp samples. In H7N1 inoculated birds, virus was detected in oropharyngeal swabs from 1 dpi to the end of the experiment; in cloacal swabs and feather pulp, viral shedding was observed from 2 dpi to 8 dpi (Figure 4A). Concerning H7N1 contact birds, detection was similar to that observed in inoculated partridges, although it started one day later (Figure 4B). Detection levels for these H7N1 inoculated animals ranged between 4 and 10 log_10 _viral RNA copies/sample, and feather pulp shedding was higher than in both oropharyngeal and cloacal swabs, particularly between 2 and 8 dpi.

Among the H7N9 infected birds, 6 out of 8 inoculated birds showed viral shedding mainly by the oropharyngeal route from 1 to 3 dpi (Figure 4C). One infected animal excreted virus by this route until 8 dpi. Only one animal shed minimal amounts of virus (2.37 log_10 _viral RNA copies/sample) by cloacal route at 1 dpi, and no viral shedding was detected in the feather pulps. Contact animals in this group did not shed virus by any of the studied routes during the whole experiment.

### Serology

H7N1 infected birds were ELISA positive from 6 dpi onwards; interestingly, 2 out of 4 seropositive partridges at 8 dpi were contact birds. On the contrary, 3 out of 8 H7N9 infected partridges showed ELISA positive results at 15 dpi. These three animals were the ones that excreted virus in a more consistent manner. None of the four contact animals in this group seroconverted, suggesting that these birds did not get infected by contact.

## Discussion

Although the red-legged partridge is one of the game bird species most frequently raised in outdoor operations, no studies had previously investigated the infection dynamics of avian influenza viruses in this species. In order to elucidate their putative role in the ecology of influenza A viruses, we evaluated the susceptibility of red-legged partridges to an infection with a HPAIV H7N1 strain (A/Chicken/Italy/5093/1999) and a LPAIV H7N9 strain (A/*Anas crecca*/Spain/1460/2008) by studying pathogenesis, transmission and viral shedding.

The high pathogenicity of this H7N1 HPAIV strain, evidenced by 100% mortality in this study, is in accordance with standardised IVPI tests for influenza viruses [15], and in agreement with those obtained in natural H7N1 HPAIV infections in chickens [21]. The only experimental infection published so far with HPAIV in partridges used an H5N1 HPAIV strain as inoculum [8]. In this experiment, 75% of mortality was observed in Chukar partridges (*Alectoris Chukar*) and MDT was shorter than in our experiment (4.5 dpi). Therefore, mortality due to infection with H7N1 in red-legged partridges seems to appear slightly later than in the H5N1 infected chicken and Chukar partridges [8]. This delay in the onset of mortality could be due to the unique virulence of the H5N1 HPAIV [22]. Clinically, progressive neurologic dysfunction, the most pronounced sign in surviving birds, correlated with the observations of Perkins and Swayne [8] in Chukar partridges. Gross lesions were observed in tissues that are known to be target organs for influenza A viruses in other gallinaceous species [8,9], such as the kidney or *fasciae *sheaths of the muscles. The general predilection of the virus for epithelia of the upper digestive, respiratory and urinary tract, pancreas and liver, feather pulp and CNS, has been extensively described in chickens infected with other HPAIV subtypes [8,23,24]. Localisation of H7N1 antigen in the parenchyma of other organs, such as the lower digestive tract, bursa of Fabricius and skeletal muscle, was less consistent and more focalised, supporting the opinion that virus distribution in the host organism is dependent on particular host factors [8].

The onset of clinical signs in H7N1 birds, both intranasally inoculated and contact animals, proved effective transmission of the virus from infected partridges to naïve contact birds. Moreover, not only inoculated birds seroconverted but also contact birds. Surprisingly, at 1 dpi all inoculated animals only showed oropharyngeal shedding, suggesting that contact birds had been infected by virus shed from the oral cavity of the inoculated animals. This finding could indicate a shift from the classical faecal-oral route to the oral-oral route (possibly through shared drinking water) in H7N1 infection, as some authors have already pointed out [2,9,25,26]. Our results suggest that in red-legged partridge, feather follicles could be a potential source for virus transmission, especially in recently dead individuals that are susceptible of feather picking. Interestingly, to date, few studies have evidenced the relevance of feathers as an important location for viral replication and potential origin of dissemination in HPAIV infection [27-29], and none of them have demonstrated the significance of this location in partridges.

The high susceptibility of partridges to H7N1 infection would make them a good sentinel species for detection of HPAIV. Since the partridges shed virus at high concentrations before death, this species could contribute to viral transmission during a local outbreak in free-living birds, in countries where partridges are found in the wild or are reared in outdoor operations. The delay between the onset of virus shedding and the appearance of clinical signs (around three days in the present experiment) could have important consequences in relation to the risk of spreading disease into the wild by releasing apparently healthy farm-reared partridges for hunting purposes. The implementation of sanitary surveillance measures prior to and after release is of importance to avoid introduction of avian influenza viruses, as well as other pathogens, in the natural ecosystem.

Our findings in H7N9 LPAIV infected birds correlate well with those obtained by Humberd et al. [6] in their experiment, in which no clinical disease was observed in ring-necked pheasants (*Phasianus colchicus*) and Chukar partridges infected with different subtypes of LPAIV. By this author, Chukar partridges were considered as short-term shedders of LPAIV, with the respiratory tract being the main viral excretion route. Likewise, in our study only limited viral shedding was detected in few inoculated birds most of which also seroconverted. Thus, our results suggest that partridges do not play a significant role as reservoir species for LPAIV, because only little, likely local, replication and short term shedding of low amounts of virus occurs in this species.

Based on our studies, firstly feather pulp, but also cloacal and oropharyngeal swabs, can be successfully used for virus detection in surveillance programs. In addition, the CNS and also pancreas and heart specimens are useful both for virus detection and histopathological diagnosis. In conclusion, although further studies with HPAIV and LPAIV strains should be performed, our observations suggest that the red-legged partridge is not likely to be a reservoir species for LPAI viruses but they are highly susceptible to H7N1 HPAIV and develop severe clinical disease and prolonged viral shedding. Thus, this species should be included in passive surveillance programs in order to prevent economical losses from HPAIV outbreaks.

## Competing interests

The authors declare that they have no competing interests.

## Authors' contributions

NB and FXA prepared the viruses used in this study. KB, EPR, RD, AC, JVA and NM participated in the daily monitoring of the clinical signs and the sampling of the animals during all the experimental period. KB, EPR, RD, AR, AC and NM performed the necropsies and the tissue sampling. RV performed the histopathology and immunohistochemistry techniques of the necropsy tissues. KB, RD and NM carried out the histopathological examinations. EPR and NB carried out the avian influenza virus quantitation by real time RT-PCR (qRRT-PCR) and the serology assays. RD, AD, FXA, MB, UH and NM conceived the study and participated in its design and coordination. All authors read and approved the final manuscript.
